# Decoding a polyamine signal to direct vascular fate: thermospermine meets the ribosome

**DOI:** 10.1093/jxb/erag257

**Published:** 2026-05-27

**Authors:** Lázara Aline Simões Silva, Munevver Dogramaci, Wagner Campos Otoni

**Affiliations:** National Institute of Science and Technology on Plant Physiology under Stress Conditions, Departamento de Biologia Vegetal/BIOAGRO, Universidade Federal de Viçosa, Viçosa, MG 36570-900, Brazil; Institute of Plant Sciences, University of Bern, Bern 3013, Switzerland; Edward T. Schafer Agricultural Research Center, USDA-Agricultural Research Service, Fargo, ND 58102, USA; National Institute of Science and Technology on Plant Physiology under Stress Conditions, Departamento de Biologia Vegetal/BIOAGRO, Universidade Federal de Viçosa, Viçosa, MG 36570-900, Brazil; University of Manchester, UK

**Keywords:** *ACAULIS5*, OVAC, ribosome modification, thermospermine, translational control, vascular development

## Abstract

The recruitment of thermospermine to OVERACHIEVER-modified ribosomes represents a substantive advance in understanding how polyamines regulate vascular differentiation during plant development.

**Polyamines are polycationic molecules involved in plant growth, stress responses, and developmental regulation. Plants primarily accumulate four polyamines: putrescine (Put), spermidine (Spd), spermine (Spm), and its isomer thermospermine (tSpm). Among these, thermospermine, plays a key role in vascular development in angiosperms. Its expression in vascular precursor cells is induced by the auxin-dependent TARGET OF MONOPTEROS 5–LONESOME HIGHWAY (LHW–TMO5) transcriptional module. Loss-of-function mutations in *ACAULIS5* (*ACL5*), encoding thermospermine synthase, cause dwarf plants with excessive xylem formation in Arabidopsis. Thermospermine negatively regulates xylem differentiation by promoting translation of *SUPPRESSOR OF ACAULIS 51 (SAC51*) family transcripts through derepression of upstream open reading frames. Recent discoveries have expanded this model by linking thermospermine signaling, ribosome function, and RNA metabolism, suggesting that regulation occurs via ribosome-mediated translation.**

## A small metabolite with outsized developmental impact

The thermospermine signaling pathway has been genetically well-characterized. In Arabidopsis, loss of the thermospermine synthase ACAULIS5 (ACL5) results in excessive xylem differentiation, vascular disorganization, and severe dwarfism, firmly establishing thermospermine as a suppressor of vessel formation (Hanzawa *et al*., 2000; Imai *et al*., 2006; Kakehi *et al*., 2008; Takahashi, 2024). Subsequent suppressor screens and reporter assays have revealed that thermospermine enhances translation of *SUPPRESSOR OF ACAULIS 51 (SAC51)* family basic helix–loop–helix transcription factors by derepressing conserved upstream open reading frames (uORFs) (Ramachandran *et al*., 2017; Ishitsuka *et al*., 2019; Mutsuda *et al*., 2025).

Yet, a fundamental mechanistic question has persisted: how can a small, diffusible polycation achieve transcript specificity *in vivo*? Polyamines bind RNA promiscuously, and are often viewed as general stabilizers of nucleic acid structure (Gupta *et al*., 2013; Bano *et al*., 2020; Alijani *et al*., 2025). The specificity required to modulate a defined developmental circuit—most notably the TARGET OF MONOPTEROS 5–LONESOME HIGHWAY (TMO5–LHW) module driving vascular proliferation and vessel differentiation—seemed difficult to reconcile with a non-specific RNA-binding metabolite (De Rybel *et al*., 2014; Ohashi-Ito and Fukuda, 2016; Xu *et al*., 2025). The field lacked a molecular ‘decoder’ that could convert thermospermine accumulation into a selective translational output.

## RNA processing and ribosome modification in thermospermine responses

The idea that ribosomes function as regulatory platforms rather than passive translation machines has gained increasing support in recent years, with Ko *et al*. (2026) finally revealing the underlying mechanism. The authors identified OVERACHIEVER (OVAC), a 25S rRNA methyltransferase responsible for methylating m^3^U2952 within the peptidyl transferase center of the ribosome. This single-base modification created a chemically distinct ribosomal state that enabled functional thermospermine binding at a defined site bridging the P-site tRNA and adjacent rRNA residues.

Two aspects of this discovery are particularly significant. First, thermospermine binding is not inferred indirectly, but is tied to a specific rRNA modification and structural context. Second, the translational output is selective rather than general. On OVAC-modified ribosomes, thermospermine enhances translation of *SAC51-LIKE (SACL)* transcripts, while repressing translation of *LHW*.

This bifunctionality has direct developmental consequences. SACL proteins compete with TMO5 for LHW binding, thereby destabilizing the TMO5–LHW heterodimer that promotes vascular proliferation and vessel differentiation. In parallel, reduced *LHW* translation further dampens the pro-differentiation module (Ohashi-Ito and Fukuda, 2016; Yamamoto and Takahashi, 2017; Nishii *et al*., 2023; Xu *et al*., 2025). Thus, a metabolite–ribosome interaction modulates both an inhibitory branch (*SACL* up-regulation) and a core regulatory node (*LHW* down-regulation) within the same developmental circuit.

This evidence indicates that thermospermine is not just a passive modulator of translation, but a metabolite decoded by chemically modified ribosomes.

## Converging evidence: ribosomal suppressors and RNA processing as gatekeepers

Importantly, this ribosome-centered model is reinforced by independent genetic evidence. Mutsuda *et al*. (2025) identified ribosomal suppressor alleles, including mutations in the genes encoding ribosomal protein L10 A (*RPL10A*) and ribosomal protein L4 A (*RPL4A*), which specifically enhanced translation of thermospermine-responsive uORF reporters, without broadly increasing translation of unrelated constructs. Such transcript-class specificity argues against a general increase in translational capacity, and instead suggests altered ribosome activity towards defined mRNA subsets.

Saraumi *et al.* (2025) reported thermospermine-insensitive mutants (*its1–its4*) mapping to RNA processing and RNA modification factors, including a SPOUT-domain methyltransferase homolog, the rRNA pseudouridine synthase CBF5 (Centromere Binding Factor 5)/NAP57 (Nucleolar protein NAP57 homolog), the spliceosome disassembly factor STIPL1 (Spliceosomal Timekeeper Locus 1)/NTR1 (NTC-Related Protein 1), and the RNA-binding protein PHIP1 (Plant Heterogeneous nuclear ribonucleoprotein I homolog Protein 1). Notably, the SPOUT-domain methyltransferase identified as ITS1 (Insensitive to Thermospermine 1) is identical to OVAC, the RNA methylation enzyme that is the focus of the present article. Furthermore, these mutants accumulate thermospermine yet fail to implement canonical phenotypic responses. Hence, thermospermine output requires a permissive RNA and ribosome environment, consistent with OVAC-dependent decoding. Together, these findings support a model, whereby thermospermine responsiveness is conditioned by ribosome composition, rRNA modification, and RNA processing dynamics.

## How does recent evidence change the thermospermine story?

The OVAC discovery does not overturn previous models; rather, it provides the missing mechanistic framework that consolidates them. The SAC51/SACL-centered genetic framework remains intact, but it is now embedded within a ribosome-dependent decoding mechanism (Ko *et al*., 2026). This refinement is conceptually important for several reasons.

**Fig. 1.**
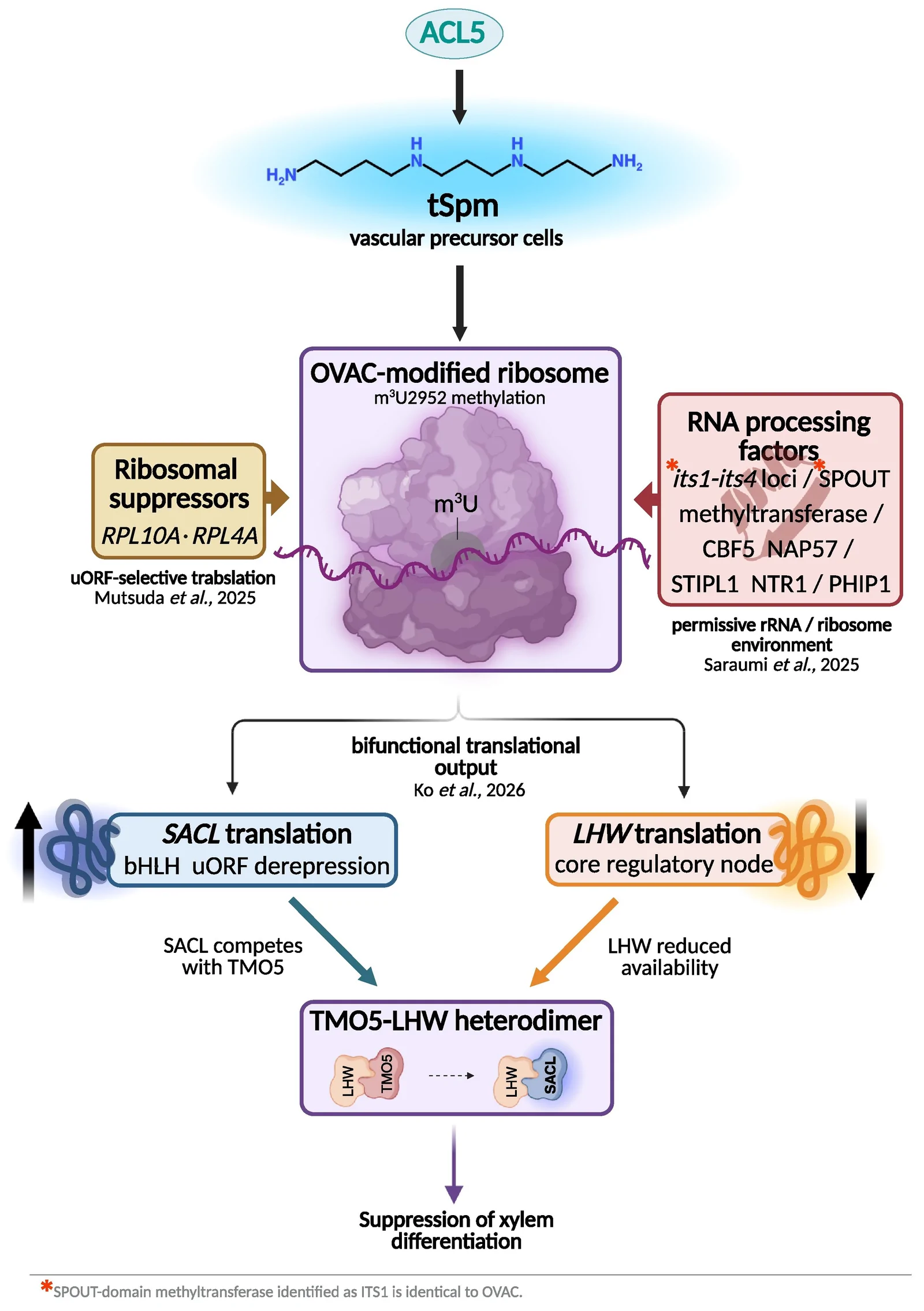
Converging genetic and molecular evidence positions ribosome composition and RNA processing as prerequisite gatekeepers of thermospermine responsiveness. Thermospermine (tSpm), synthesized by ACL5 in vascular precursor cells, is decoded by OVAC-methylated ribosomes carrying the m^3^U2952 modification at the peptidyl transferase center. Two independent lines of evidence reinforce this ribosome-centric model. First, suppressor alleles of *RPL10A* and *RPL4A* (Mutsuda *et al*., 2025) selectively enhance translation of thermospermine-responsive uORF-containing transcripts without broadly increasing translational output, indicating that ribosome composition modulates access to defined mRNA subsets. Second, thermospermine-insensitive mutants (*its1–its4*; Saraumi *et al*., 2025), which accumulate normal levels of thermospermine yet fail to execute canonical responses, map to RNA processing and modification factors—including a SPOUT-domain methyltransferase (*ITS1*, identical to OVAC), the rRNA pseudouridine synthase CBF5/NAP57, the spliceosome disassembly factor STIPL1/NTR1, and the RNA-binding protein PHIP1—establishing that a permissive RNA and ribosome environment is required upstream of signal interpretation. On OVAC-modified ribosomes, thermospermine exerts a bifunctional translational output (Ko *et al*., 2026): it simultaneously up-regulates *SACL* family transcripts (via uORF derepression) and down-regulates *LHW* translation. SACL proteins compete with TMO5 for LHW binding, and reduced LHW availability further dampens the pro-differentiation TMO5–LHW heterodimer, ultimately suppressing xylem differentiation and limiting vascular proliferation. Created in BioRender. Silva, L.A. S. (2026) https://BioRender.com/qihsb6i.

First, it strengthens the causal link between polyamine metabolism and vascular patterning (Kakehi *et al*., 2008; Ramachandran *et al*., 2017; Bano *et al*., 2020). Instead of invoking indirect or poorly defined translational effects, the pathway now includes a discrete molecular step: thermospermine is interpreted by a chemically modified ribosome. Second, it reframes tissue specificity. Spatial expression of *ACL5* has long been considered central to thermospermine function (Vera-Sirera *et al*., 2015; Ishitsuka *et al*., 2019; Takahashi, 2024). The new model suggests that ribosome heterogeneity—specifically the distribution of OVAC-dependent m^3^U2952-modified ribosomes—may be equally important to context dependence (Fig. 1). Cell-type specificity could therefore depend on both metabolite production and ribosome chemistry. Third, it places plant vascular development within a broader conceptual framework, in which rRNA modifications influence translational selectivity. Ribosome heterogeneity has been discussed extensively in animal systems (Genuth and Barna, 2018; Aspden *et al*., 2025), yet its developmental relevance in plants has remained less clear. Thermospermine now provides a concrete case, whereby a specific rRNA modification mediates a metabolite-dependent developmental decision.

## Concluding perspectives

The recruitment of thermospermine to OVAC-modified ribosomes represents a substantive advance in understanding how polyamines influence plant development. By identifying a chemically defined decoding mechanism, this research transforms thermospermine from a somewhat enigmatic translational modulator into a metabolite with a known developmental-specific mechanism.

More broadly, it highlights ribosome chemistry as an underexplored regulatory layer in plant pattern formation (Box 1). If rRNA modification landscapes help determine where metabolites are ‘read,’ then developmental decisions may be shaped as much by ribosome state as by transcriptional networks. Thermospermine may therefore serve as a paradigm for how metabolic cues interface with translational control to drive plant form.

Box 1. **Emerging priorities for the field**

Recent discoveries expand the role of thermospermine, from a specialized polyamine pathway to a test case for metabolite–ribosome interactions as regulators of plant development. The possibility that thermospermine acts in a cell-dependent manner raises questions about transport routes, source–sink relationships, and the evolutionary rationale behind its role limited to the regulation of xylem formation.

- Where and when are m^3^U2952-modified ribosomes enriched? Spatially resolved mapping of OVAC activity in procambium, cambium, and differentiating xylem could clarify how decoding capacity varies across vascular domains.
- What defines the broader thermospermine-sensitive translational regulon? While *SAC51/SACL* transcripts are the best-characterized targets, ribosome profiling suggests additional candidates. Cell-type–resolved approaches will be necessary to determine whether distinct vascular states harbor distinct decoding landscapes.
- How does thermospermine binding at the peptidyl transferase site generate opposite outputs for SACL and LHW? Dissecting uORF architecture, peptide-dependent ribosome stalling, and reinitiation competence will be critical for predictive modeling.
